# Assessing the host range of *Anastatus orientalis*, an egg parasitoid of spotted lanternfly (*Lycorma delicatula)* using Eastern U.S. non-target species

**DOI:** 10.3389/finsc.2023.1154697

**Published:** 2023-04-18

**Authors:** Hannah J. Broadley, Steven J. Sipolski, Danielle B. Pitt, Kim A. Hoelmer, Xiao-yi Wang, Liang-ming Cao, Lisa A. Tewksbury, Tyler J. Hagerty, Charles R. Bartlett, Alana D. Russell, Yunke Wu, Shannon C. Davis, Joe M. Kaser, Joseph S. Elkinton, Juli R. Gould

**Affiliations:** ^1^ Forest Pest Methods Laboratory, United States Department of Agriculture, Animal and Plant Health Inspection Service, Plant Protection and Quarantine, Science and Technology, Buzzards Bay, MA, United States; ^2^ Department of Environmental Conservation, University of Massachusetts, Amherst, MA, United States; ^3^ Beneficial Insects Introduction Research Unit, United States Department of Agriculture, Agricultural Research Service, Newark, DE, United States; ^4^ Key Laboratory of Forest Protection of National Forestry and Grassland Administration, Ecology and Nature Conservation Institute, Chinese Academy of Forestry, Beijing, China; ^5^ Department of Plant Sciences and Entomology, University of Rhode Island, Kingston, RI, United States; ^6^ Department of Entomology and Wildlife Ecology, University of Delaware, Newark, DE, United States; ^7^ Department of Ecology and Evolutionary Biology, Cornell University, Ithaca, NY, United States

**Keywords:** biological control, Eupelmidae, Fulgoridae, invasive species, natural enemy

## Abstract

The spotted lanternfly, *Lycorma delicatula* (Hemiptera: Fulgoridae), an invasive planthopper discovered in Pennsylvania, U.S. in 2014, has spread to many surrounding states despite quarantines and control efforts, and further spread is anticipated. A classical (importation) biological control program would contribute to the long-term management of *L. delicatula* in the eastern U.S. In its native range of China, *Anastatus orientalis* (Hymenoptera: Eupelmidae), an egg parasitoid, causes significant mortality. *Anastatus orientalis* consists of multiple haplotypes that differ in important biological parameters. To delineate the physiological host range of *A. orientalis* Haplotype C, we completed no-choice and choice testing. No-choice testing of non-target eggs from 36 insect species spanning six orders and 18 families showed that physiologically this haplotype of *A. orientalis* can develop in a variety of host species eggs from the families Coreidae, Fulgoridae, Pentatomidae, and Saturniidae. Ten of the 16 species that were attacked in the no-choice tests were also attacked in the choice tests. The production of progeny on non-target egg masses was significantly lower than on the controls (*L. delicatula* egg masses run simultaneously) in the no-choice and choice tests. For the non-target species that were attacked and resulted in female wasp progeny, these females were able to produce their own progeny at the same rate as control females that were reared from the *L. delicatula* eggs. Larger host eggs corresponded to an increased female-biased sex ratio of the progeny, suggesting that gravid females select them for fertilized eggs. Results from these studies suggest that *A. orientalis* Haplotype C prefers to parasitize *L. delicatula* egg masses but is capable of developing in some non-target species.

## Introduction

The spotted lanternfly, *Lycorma delicatula* (White) (Hemiptera: Fulgoridae), is an invasive planthopper first detected in Berks County Pennsylvania in the fall of 2014 (1). It has since spread extensively within Pennsylvania and neighboring states where it threatens the grape, hops, tree fruit, plant nursery, and timber industries (2). Quarantines have been put in place to restrict the movement of plant, wood, and stone products, but because egg masses are cryptic and it is difficult to regulate the movement of all items potentially harboring them (3, 4), the pest has continued to spread and now has established populations in 14 states plus reports in two more (5). Research on host plant utilization indicates that *L. delicatula* can develop to the adult stage on several host plant species in addition to its favored host, tree-of-heaven (*Ailanthus altissima* (Mill.) Swin) (6), which further confounds the eradication efforts. Some mortality from resident fungal pathogens (7), egg parasitoids (8), and predation (9) have been noted but the incidences are rare and so far have resulted in inconsequential mortality rates. However, in its native range in China, several parasitoid wasps attack *L. delicatula* eggs and nymphs and cause significant mortality (10, 11). A classical (or importation) biological control program could contribute to management efforts against *L. delicatula*.

*Anastatus orientalis* Yang & Choi (Hymenoptera: Eupelmidae) is a small parasitic wasp that attacks *L. delicatula* eggs in its native range in China (10), most commonly in northeastern China (11). In Chinese field collections, *A. orientalis* parasitizes at a relatively high rate, ranging from 20 to 80% of egg masses attacked and up to 40% of eggs parasitized within individual egg masses (11, 12). Additionally, while suitable host plants are abundant for *L. delicatula* in these same locations in China, populations of *L. delicatula* are low compared to those of the invasive population in the U.S (11). This suggests that *A. orientalis* and other natural enemies are having a strong effect on *L. delicatula* populations in China. *Anastatus orientalis* was selected as a candidate biological control agent for helping to manage *L. delicatula* in South Korea where it is also invasive (13, 14). In 2011, the South Korean Rural Development Administration National Institute of Agricultural Sciences initiated a collaboration with the Chinese Academy of Forestry to collect and evaluate *A. orientalis* as a candidate biological control agent for invasive *L. delicatula* in Korea (10, 15, 16) and introductions of the parasitoids were made soon afterwards. *Anastatus orientalis* is being considered as a candidate biological control agent for the invasive populations of *L. delicatula* in the eastern United States.

Evaluation of the physiological host range is an essential first step to determine whether a natural enemy will be deemed sufficiently host-specific (17, 18) to be suitable for release as a biological control agent. We now know that multiple haplotypes of *A. orientalis* are present in China (19). Therefore, while initial testing work conducted for the releases in Korea suggested that the haplotype of *A. orientalis* released in Korea did not attack their species of concern (15), it is essential to test the strain of *A. orientalis* maintained in U.S. quarantine cultures (20) against the eggs of selected non-target species present in the United States.

Here we outline our results from no-choice and choice tests in which we tested eggs of planthoppers, stink bugs, other hemipterans, silk moths and selected other species that are native to or resident in the United States as potential hosts of *A. orientalis*. This manuscript describes testing of species of concern present in the eastern United States where *L. delicatula* currently is invasive. Due to concern about the threat of invasion by *L. delicatula* to the western United States, simultaneous coordinated testing of species resident to the western coast of the United States was conducted at the University of California Riverside Insectary and Quarantine Facility (21). To evaluate the physiological host range, we first exposed non-target eggs to mated female *A. orientalis* wasps in no-choice tests where the wasps had access to the non-target species eggs for a full week of exposure. For any species that were parasitized in the no-choice tests, we then conducted choice tests. For choice tests, the parasitoid was offered the target host, *L. delicatula*, together with the non-target egg masses so that the wasps could choose which (or both) host(s) to parasitize. This provided information on behavior and showed whether a parasitoid would choose to use a non-target when the primary host was also available. We present results of testing more than 30 different non-target species. Testing included *Poblicia fuliginosa* (Olivier) (Hemiptera: Fulgoridae), which is a closely related native species present in the current invasive range of *L. delicatula*. Initial priority species also included other species corresponding to taxonomic relatedness, the morphological similarity of egg masses, and occurrence in the same microhabitat as *L. delicatula* in the landscape.

## Materials and methods

### Parasitoid colony

The parasitoid colony we tested is maintained in the containment facility at the USDA APHIS Forest Pest Methods Laboratory, Buzzards Bay, MA, and all studies were completed in this facility. Laboratory colonies of *A. orientalis* were established from parasitized egg masses collected annually from the field in Beijing, China (N39.9925, E116.2109) from 2016 to 2019. To confirm the species and haplotype, we extracted genomic DNA from a single leg pulled from five randomly selected colony specimens using the QIAGEN DNeasy Blood & Tissue Kit (QIAGEN, Germantown, MD). Each specimen was genotyped for two mitochondrial fragments of the COI gene. We aligned the newly generated DNA sequences with reference sequences from the six haplotype groups identified in Wu et al. (19). Two months later, we conducted a second round of genotyping with 40 specimens to ensure colony homogeneity. After another eight months, we conducted the final round of genotyping including eight more specimens from the C colony and another 60 from the other haplotype colonies to ensure no contamination between strains.

We reared the wasps using *L. delicatula* egg masses collected each year from December to March in Pennsylvania. We collected egg masses whole and intact by cutting them from bark (primarily from *A. altissima*) and held them in a growth chamber at a constant 5°C with no light. We maintained the *A. orientalis* laboratory colony by setting up groups of three to five females with one or two males in a medium-sized rearing container (473-ml plastic deli cup, AD16 GenPak, Charlotte, NC) with the following temperature and light conditions: daily high of 25°C and low of 14°C, lights on 5:55 AM to 6:23 PM, 65% RH. These conditions were chosen to emulate environmental conditions in mid-September in Beijing, China, and hereafter will be referred to as ‘Beijing-fall conditions’ and work well for continuous rearing of this haplotype of *A. orientalis* (20). We provided each set of wasps with a streak of honey as a food source. The wasps were held without access to egg masses for one week (corresponding with their preoviposition period), and then given one *L. delicatula* egg mass for a period of one week. We held the developing progeny under Beijing-fall conditions for another month then moved to a 25°C constant temperature and a light cycle of 16 hours light and 8 hours dark to promote emergence. Further rearing details are provided in Broadley et al. (20).

### Non-target selection, collection, and rearing

We selected non-target species for testing based on relatedness to *L. delicatula* (prioritizing *Poblicia fuliginosa*, which is in the same family, Fulgoridae) and other planthoppers, morphological similarity of egg clusters, and based on prior information on species utilized by other *Anastatus* species (22–25). Potential eastern U.S. non-target species spanning six insect orders and 18 families were field collected or acquired from laboratory colonies for no-choice and choice testing (**Table 1**). We saved voucher specimens of all species. We acquired species not already established in laboratory colonies as nymphs and adults through field collection in natural habitats, using visual inspection of host plant material, sweep netting, or beating of host plant material. We then moved field collected insects into rearing conditions designed for each species’ needs to produce egg masses. We kept phytophagous species on whole potted host plants or provided plant material such as vegetables. Typical laboratory rearing conditions were set at a temperature of 25°C and a light cycle of 16 hours light and 8 hours dark. We maintained entomophagous species in enclosures mimicking their natural habitat and provisioned them with insects that met their dietary requirements.

**Table 1:** Non-target species collection information.

Order	Family	Species	Collection location/Commercially obtained	Acquisition date
Blattodea	Blaberidae	*Nauphoera cinerea*	Reared by Alex Baranowski in colony	Jul. 26, 2020
Coleoptera	Coccinellidae	*Harmonia axyridis*	Kingston, Charlestown, Exeter, Hopkinton, RI/Bristol & Southbury, CT	Nov. 2019
Coleoptera	Coccinellidae	*Hippodamia convergens*	Purchased live adults from Natures Good Guys	Feb. 9, 2021
Hemiptera	Acanaloniidae	*Acanalonia bivittata*	Exeter, RI and University of Delaware Farm, Newark, DE	Aug.-Sep. 2020 (RI); Jul. and Aug. 2017 - 2022 (DE)
Hemiptera	Acanaloniidae	*Acanalonia conica*	Field-caught individuals University of Delaware Farm, Newark, DE added to colony	Jul. and Aug. 2017 - 2022
Hemiptera	Coreidae	*Anasa armigera*	Kingston, RI	Aug. 2019
Hemiptera	Coreidae	*Anasa tristis*	Kingston, RI	Aug. 2020
Hemiptera	Dictyopharidae	*Rhynchomitra microrhina*	Field-caught from the University of Delaware Farm, Newark, DE added to colony	Jul. and Aug. 2017 - 2022
Hemiptera	Flatidae	*Flatormenis proxima*	Field-caught from the University of Delaware Farm, Newark, DE added to colony	Jul. and Aug. 2017 - 2022
Hemiptera	Fulgoridae	*Poblicia fuliginosa*	Field-caught from Jones Lake, Bladen Co., NC	Aug. 2018 - 2022
Hemiptera	Lygaeidae	*Oncopeltus fasciatus*	Kingston, RI & Bristol, CT	Aug. 2020
Hemiptera	Membracidae	*Thelia bimaculata*	Newark, New Castle Co., DE	Jun. and Jul. 2020-2021
Hemiptera	Pentatomidae	*Chinavia hilaris*	Kingston, North Kingstown, RI/Windsor, Falls Village, CT; and <50 km from Newark, DE	Jun.-Sep. 2018 and 2020
Hemiptera	Pentatomidae	*Edessa florida*	<50 km from Newark, DE	Jun.-Sep. 2018 and 2020
Hemiptera	Pentatomidae	*Euschistus servus*	Kingston & Exeter, RI	Jun.-Sep. 2020
Hemiptera	Pentatomidae	*Euschistus tristigmus*	<50 km from Newark, DE	Jun.-Sep. 2019 - 2021
Hemiptera	Pentatomidae	*Halyomorpha halys*	NJ Dept. of Agriculture egg masses reared in Newark, DE	Continuously reared
Hemiptera	Pentatomidae	*Murgantia histrionica*	<50 km from Newark, DE	Jun.-Sep. 2019 - 2021
Hemiptera	Pentatomidae	*Oebalus pugnax*	<50 km from Newark, DE	Jun.-Sep. 2020
Hemiptera	Pentatomidae	*Podisus maculiventris*	<50 km from Newark, DE	Jun.-Sep. 2019 and 2020
Hemiptera	Pentatomidae	*Thyanta custator*	<50 km from Newark, DE	Jun.-Sep. 2020
Hemiptera	Reduviidae	*Phymata pennsylvanica*	Exeter, RI/Southbury and Bristol, CT	Aug. 2020
Hemiptera	Reduviidae	*Zelus luridus*	Kingston, RI/Bristol, CT	Jun.-Sep. 2020
Lepidoptera	Bombycidae	*Bombyx mori*	Reared by Alex Baranowski in colony	Jan. 8, 2020
Lepidoptera	Erebidae	*Lymantria dispar dispar*	From the Forest Pest Methods Laboratory, Buzzards Bay, MA continuous colony	Sep. 2019
Lepidoptera	Lasiocampidae	*Malacosoma americanum*	New Shoreham, RI; Exeter, RI: Bristol, CT	Dec. 2019 and Mar. 2020
Lepidoptera	Nymphalidae	*Danaus plexippus*	Kingston, Charlestown, Exeter, RI	Jul. and Aug. 2020
Lepidoptera	Saturniidae	*Actias luna*	Purchased cocoons from Carolina Biological and Magic Wings Butterflies	Dec. 2020; Feb. 2021; Feb 2022
Lepidoptera	Saturniidae	*Antheraea polyphemus*	Purchased cocoons from Carolina Biological and Magic Wings Butterflies	Dec. 7-14, 2020
Lepidoptera	Saturniidae	*Callosamia promethea*	Reared by Kathrine Straley	Aug. 2020
Lepidoptera	Saturniidae	*Hyalophora cecropia*	Purchased cocoons from Carolina Biological and Magic Wings Butterflies	Dec. 7-14, 2020
Mantodea	Mantidae	*Mantis religiosa*	Kingston and Exeter, RI	Jul. and Aug. 2020
Mantodea	Mantidae	*Stagmomantis carolina*	Brooklyn, NY	Jan. 7, 2020
Mantodea	Mantidae	Stagmomantis limbata*	Davis, CA	Jan. 2020
Mantodea	Mantidae	*Tenodera sinensis*	Kingston and W. Greenwich, RI/Southington, CT	Sep. and Oct. 2019
Phasmatodea	Diapheromeridae	*Manomera blatchleyi*	Kingston, RI	Jul. 2020

*Not resident to the eastern United States.

### No-choice host testing experiments

To prepare for no-choice testing, we gently aspirated recently emerged *A. orientalis* wasps (24 hours old or less) from their plastic rearing containers. We placed up to five female and five male wasps together in a 1:1 ratio in a small glass rearing container (8 oz wide mouth mason jar 00500, Kerr, Newell, Atlanta, GA). The jars were streaked with honey for food and covered with mesh (no-see-um polyester netting 7250NSW, Bioquip, Rancho Dominguez, CA) to provide ventilation. Before use in experiments, the glass containers were autoclaved, and together with the metal lid rings and mesh, were washed with a one percent Citronox (Alcanox, White Plains, NY) solution, rinsed with DI water, then acetone, and air dried. This cleaning removed chemical traces that might affect the olfactory cues presented to the wasp because previous work found that *A. orientalis* responded to chemical traces left behind by *L. delicatula* (25). We placed the rearing containers in secondary plastic containers (6 Quart Storage Box 1642, Sterilite, Townsend, MA) to further isolate the tests from any outside olfactory cues. Before use, the secondary containers were also washed in a 1% Citronox solution and rinsed with DI water, then rinsed with 95% ethanol and left to air dry. The wasps were given a one-week preoviposition period at Beijing-fall conditions (described above).

Following the one-week preoviposition period, we removed all male wasps, and the female wasps were moved individually to a new glass rearing container and given either a non-target egg mass or a *L. delicatula* egg mass as a control to parasitize. The non-target eggs were put into testing as young as possible with the aim of testing them within less than a week. If they were not run within a five-day window, they were ramped down by moving them into 10°C for half a week then into 5°C until they could be put into testing. When multiple non-target species were tested during the same day, a single set of controls for that day was used for all the simultaneously-tested species, with sufficient numbers of control replicates to match the number of replicates of the non-target species having the most replication on that day. We tested non-target and control host species in separate secondary plastic containers to avoid mixing kairomones and other chemical cues. All wasps were allowed one week of oviposition under Beijing-fall conditions. After the one week, we removed the female wasps from the egg masses and preserved them in 95% ethanol. We placed the egg masses individually in plastic rearing containers (6oz, Clear Hinged Deli Cup, AD06, GenPak, Charlotte, NC, modified to include a mesh lid) and held them in Beijing-fall conditions for one month and then subsequently placed them under 25°C long day conditions for emergence. Our goal was to test 30 replicates for each non-target species, but some of the species were challenging to obtain so less replication was possible. We recorded host nymphal and F1 parasitoid emergence daily (Monday-Friday) until there was no further emergence for one month. We noted the sex of the parasitoids that emerged. Non-target nymphs were saved in 95% ethanol for vouchers.

### Egg size measurements

We recorded egg size measurements for each species, including *L. delicatula*, to determine the mean volume. We measured ten eggs from three of the egg masses being dissected. For circular eggs, we measured the diameter; for ellipsoid and cube-shaped eggs, we measured height, width, and length; for cylindrical and oblate spheroid eggs, we measured width and height; and lastly for pentagonal frustum-shaped eggs, we measured the egg height, the width at the top of the pentagon, and width at the base of the pentagon. The volume of the eggs was calculated from these measurements.

We dissected a subset of available replicates (**Table 2**) for each non-target species and a comparative subset of *L. delicatula* egg masses. We waited at least two weeks after the last parasitoid emergence before dissecting the egg masses. For egg masses with no parasitoid emergence, we dissected them after one month. For those dissected, we recorded the fate of each egg.

**Table 2:** Egg masses dissected from no-choice testing.

Order	Family	Species	Number of replicates dissected	Detection of any unsuccessful *A. orientalis* emergence	Mean (± SE) unsuccessful *A. orientalis* emergence per egg mass	Mean (± SE) number of eggs per egg mass	Proportion of unsuccessful *A. orientalis* emergence
Coleoptera	Coccinellidae	*Harmonia axyridis*	1	No	0	0	0
Hemiptera	Acanaloniidae	*Acanalonia bivittata*	29	No	0	0	0
Hemiptera	Acanaloniidae	*Acanalonia conica*	56	No	0	0	0
Hemiptera	Coreidae	*Anasa armigera*	28	Yes	0.07 ± 0.05	10.46 ± 0.93	0.01
Hemiptera	Coreidae	*Anasa tristis*	40	Yes	5.35 ± 1.56	25.90 ± 2.03	0.21
Hemiptera	Flatidae	*Flatormenis proxima*	8	No	0	0	0
Hemiptera	Fulgoridae	*Poblicia fuliginosa*	22	Yes	4.45 ± 1.31	32.32 ± 1.65	0.14
Hemiptera	Fulgoridae	*Lycorma delicatula*	66	Yes	3.05 ± 0.60	39.79 ± 3.05	0.08
Hemiptera	Lygaeidae	*Oncopeltus fasciatus*	11	No	0	0	0
Hemiptera	Pentatomidae	*Chinavia hilaris*	20	Yes	0.45 ± 0.30	22.55 ± 2.04	0.02
Hemiptera	Pentatomidae	*Euschistus servus*	39	Yes	0.59 ± 0.26	20.15 ± 1.70	0.03
Hemiptera	Pentatomidae	*Euschistus tristigmus*	16	No	0	0	0
Hemiptera	Pentatomidae	*Halyomorpha halys*	36	Yes	0.64 ± 0.24	25.14 ± 0.64	0.03
Hemiptera	Pentatomidae	*Murgantia histrionica*	43	Yes	0.21 ± 0.10	12.49 ± 0.84	0.02
Hemiptera	Pentatomidae	*Oebalus pugnax*	2	No	0	0	0
Hemiptera	Pentatomidae	*Podisus maculiventris*	15	No	0	0	0
Hemiptera	Pentatomidae	*Thyanta custator*	19	Yes	0.21 ± 0.21	28.10 ± 2.60	0.01
Hemiptera	Reduviidae	*Phymata pennsylvanica*	2	No	0	0	0
Hemiptera	Reduviidae	*Zelus luridus*	10	No	0	0	0
Lepidoptera	Bombycidae	*Bombyx mori*	10	No	0	0	0
Lepidoptera	Lasiocampidae	*Malacosoma americanum*	6	No	0	0	0
Lepidoptera	Nymphalidae	*Danaus plexippus*	12	No	0	0	0
Lepidoptera	Saturniidae	*Actias luna*	40	Yes	0.93 ± 0.49	30.30 ± 1.17	0.03
Lepidoptera	Saturniidae	*Antheraea polyphemus*	19	Yes	0.16 ± 0.09	23.89 ± 1.34	0.01
Lepidoptera	Saturniidae	*Hyalophora cecropia*	10	Yes	2.70 ± 2.59	27.00 ± 1.18	0.10
Mantodea	Mantidae	*Mantis religiosa*	10	No	0	0	0
Mantodea	Mantidae	*Stagmomantis carolina*	11	No	0	0	0
Mantodea	Mantidae	*Stagmomantis limbata*	11	No	0	0	0
Mantodea	Mantidae	*Tenodera sinensis*	10	No	0	0	0
Phasmatodea	Diapheromeridae	*Manomera blatchleyi*	10	No	0	0	0

The species that contained unsuccessfully emerged *A. orientalis* are indicated in gray.

### Fitness of *A. orientalis* reared from non-target eggs

For each non-target egg mass that had female wasp emergence, we evaluated the fecundity of a randomized subset of these progeny (F1 generation). We placed up to five female wasps in a plastic rearing cup. Five male wasps from a different egg mass of the same species host were added to the rearing cup for a 1:1 ratio. If five males were not available, as many as possible were added, and in the event of no male wasps being present, five male wasps produced from a *L. delicatula* egg mass were added to ensure that the females could mate. We provisioned them with honey and held them under Beijing-fall conditions for a one-week preoviposition period. Following preoviposition, we removed all male wasps and all except two female wasps. The remaining two female wasps were each placed in a container and given a single *L. delicatula* egg mass. After one week of oviposition under Beijing-fall conditions, we removed the female wasps and saved them in 95% ethanol. We allowed the egg masses to develop for one month under Beijing-fall conditions before we moved them to 25°C long day conditions for emergence. We recorded nymphal and parasitoid emergence (F2 generation) daily (Monday-Friday) and saved all female wasps in 95% ethanol.

We compared the size of female progeny reared out of non-target hosts (F1 from non-targets) as compared to the simultaneously run controls reared from *L. delicatula* (F1 from controls) and to progeny produced when these non-target reared females (F1 from non-targets) were given *L. delicatula* to parasitize (F2 from the non-target females). We measured both hind tibiae from up to 10 F1 and F2 generation female parasitoids from each category from the testing of the non-targets *Actias luna* (L.) and *Halyomorpha halys* (Stål). Hind tibia measurements can be a useful proxy for fecundity, mating ability, and longevity (26), which together can suggest greater fitness (27–29). We chose progeny reared from these two non-target species to capture a wide range of egg volumes because *A. luna* had the largest non-target egg, and *H. halys* was among the smallest non-target species to produce female progeny. For each replicate, we removed both hind tibiae from female wasps and took measurements using Leica Microsystems model M125 C dissecting microscope with LASX software Version 3.7.2.22383.

### Choice host testing experiments

For non-target species that produced wasps in the no-choice tests, we then conducted choice testing. We followed the same procedure as for the no-choice testing except that larger (16 oz wide mouth mason jar 1440061180, Ball, Newell, Atlanta, GA) glass rearing jars were used, and both the non-target and target (*L. delicatula*) egg mass were provided for oviposition. Following wasp exposure, we placed each egg mass into individual plastic rearing cups so that emergence could be recorded separately. We conducted host specificity testing between September 2018 and June 2021, with no-choice testing starting in September 2018 and choice testing starting in August 2020.

### Statistical analyses

To test for the effect of non-target as compared to the controls, we used a Wilcoxon paired-sample test with the no-choice and choice tests analyzed separately. To test the effect of egg size on the resulting sex of wasp progeny produced, we ran a generalized linear model with a logit link function and a binomial distribution. To test for a difference in the number of F2 progeny produced from each F1 female, we used a one-way ANOVA. To compare the mean tibia measurements of female *A. orientalis* wasps (F1 generation) reared from a large non-target host (*A. luna*) and a small non-target host (*H. halys*) as compared to the controls (*L. delicatula*) and the size of the female progeny of these females, we averaged the measurements of both hind tibia for each individual, then ran a one-way ANOVA and a Tukey-Kramer’s test. All statistics were run using JMP 13.1.0 (SAS Institute Inc.) and figures were constructed using JMP 13.1.0 and R version 4.1.1 (The R Foundation for Statistical Computing).

## Results

### Parasitoid haplotype

Three rounds of genotyping on 53 A*. orientalis* specimens from the wasp colony used for this study over a 10-month period showed that the colony was composed of a homogenous population all representing the same species and the same haplotype. All sequences were identical and matched with *A. orientalis* Haplotype C.

### No-choice and choice host-testing experiments

We completed no-choice testing of non-target eggs from 36 insect species spanning six orders and 18 families (**Table 3**). These tests included planthoppers (including a Fulgoridae) but also tests of non-targets from more distantly related species. Of these 36 species, *A. orientalis* was able to parasitize the eggs of 16 species and produce F1 progeny. No progeny were produced from any egg masses tested from species in the order Blattodea (cockroaches), Coleoptera (beetles), Mantodea (mantises) or Phasmatodea (stickbugs). However, every species tested in no-choice testing in the families Coreidae (leaf-footed bugs), Fulgoridae (lanternflies), Pentatomidae (stinkbugs), and Saturniidae (giant silk moths) was attacked to some degree.

**Table 3:** Non-target species tested in no-choice and choice tests.

Order	Family	Species	Common name	No-Choice Tests			Choice Tests		
				Number of replicates	*A. orientalis* Progeny	Prop. of progeny female*	Number of replicates	*A. orientalis* Progeny	Prop. of progeny female*
Blattodea	Blaberidae	*Nauphoeta cinerea*	speckled cockroach	30	no	N/A	N/A	N/A	N/A
Coleoptera	Coccinellidae	*Harmonia axyridis*	harlequin ladybird	3	no	N/A	N/A	N/A	N/A
Coleoptera	Coccinellidae	*Hippodamia convergens*	convergent ladybeetle	30	no	N/A	N/A	N/A	N/A
Hemiptera	Acanaloniidae	*Acanalonia bivittata*	two-striped planthopper	33	no	N/A	16	no	N/A
Hemiptera	Acanaloniidae	*Acanalonia conica*	green conehead planthopper	60	no	N/A	15	no	N/A
Hemiptera	Coreidae	*Anasa armigera*	horned squash bug	60	yes	0	30	yes	0
Hemiptera	Coreidae	*Anasa tristis*	squash bug	55	yes	0	30	no	N/A
Hemiptera	Dictyopharidae	*Rhynchomitra microrhina*	planthopper	1	no	N/A	N/A	N/A	N/A
Hemiptera	Flatidae	*Flatormenis proxima*	northern flatid planthopper	30	no	N/A	N/A	N/A	N/A
Hemiptera	Fulgoridae	*Poblicia fuliginosa*	sooty planthopper	33	yes	0.22	30	no	N/A
Hemiptera	Lygaeidae	*Oncopeltus fasciatus*	large milkweed bug	22	no	N/A	N/A	N/A	N/A
Hemiptera	Membracidae	*Thelia bimaculata*	locust treehopper	8	no	N/A	N/A	N/A	N/A
Hemiptera	Pentatomidae	*Chinavia hilaris*	green stink bug	15	yes	0.02	11	yes	0
Hemiptera	Pentatomidae	*Edessa florida*	Edessa stink bug	2	yes	0	0	No data	No data
Hemiptera	Pentatomidae	*Euschistus servus*	brown stink bug	30	yes	0	30	yes	0
Hemiptera	Pentatomidae	*Euschistus tristigmus*	dusky stink bug	30	yes	0	30	yes	0
Hemiptera	Pentatomidae	*Halyomorpha halys*	brown marmorated stink bug	30	yes	0.01	30	yes	0.02
Hemiptera	Pentatomidae	*Murgantia histrionica*	harlequin bug	48	yes	0	12	yes	0
Hemiptera	Pentatomidae	*Oebalus pugnax*	rice stink bug	2	yes	0	0	No data	No data
Hemiptera	Pentatomidae	*Podisus maculiventris*	spined soldier bug	35	yes	0	30	no	N/A
Hemiptera	Pentatomidae	*Thyanta custator*	red shouldered stink bug	30	yes	0	27	yes	0
Hemiptera	Reduviidae	*Phymata pennsylvanica*	Pennsylvania ambush bug	2	no	N/A	N/A	N/A	N/A
Hemiptera	Reduviidae	*Zelus luridus*	pale green assassin bug	30	no	N/A	11	no	N/A
Lepidoptera	Bombycidae	*Bombyx mori*	domestic silk moth	30	no	N/A	N/A	N/A	N/A
Lepidoptera	Erebidae	*Lymantria dispar dispar*	spongy moth	30	no	N/A	N/A	N/A	N/A
Lepidoptera	Lasiocampidae	*Malacosoma americanum*	eastern tent caterpillar	10	no	N/A	N/A	N/A	N/A
Lepidoptera	Nymphalidae	*Danaus plexippus*	monarch butterfly	30	no	N/A	N/A	N/A	N/A
Lepidoptera	Saturniidae	*Actias luna*	luna moth	36	yes	0.41	30	yes	0.61
Lepidoptera	Saturniidae	*Antheraea polyphemus*	polyphemus moth	36	yes	0.75	29	yes	0.74
Lepidoptera	Saturniidae	*Callosamia promethea*	promethea silk moth	9	yes	0	0	No data	No data
Lepidoptera	Saturniidae	*Hyalophora cecropia*	cecropia moth	30	yes	0.67	31	yes	0.6
Mantodea	Mantidae	*Mantis religiosa*	European mantis	27	no	N/A	N/A	N/A	N/A
Mantodea	Mantidae	*Stagmomantis carolina*	Carolina mantis	23	no	N/A	6	no	N/A
Mantodea	Mantidae	*Stagmomantis limbata*	bordered mantis	28	no	N/A	N/A	N/A	N/A
Mantodea	Mantidae	*Tenodera sinensis*	Chinese mantis	30	no	N/A	N/A	N/A	N/A
Phasmatodea	Diapheromeridae	*Manomera blatchleyi*	Blatchley walking stick	30	no	N/A	N/A	N/A	N/A

*The corresponding proportion of the progeny that were female when testing *L. delicatula* in the no-choice tests was 0.75 and for the choice testing was 0.78.

The egg species that produced *A. orientalis* progeny from no-choice and choice tests are indicated in gray.N/A, Not applicable.

**Figure 1:**
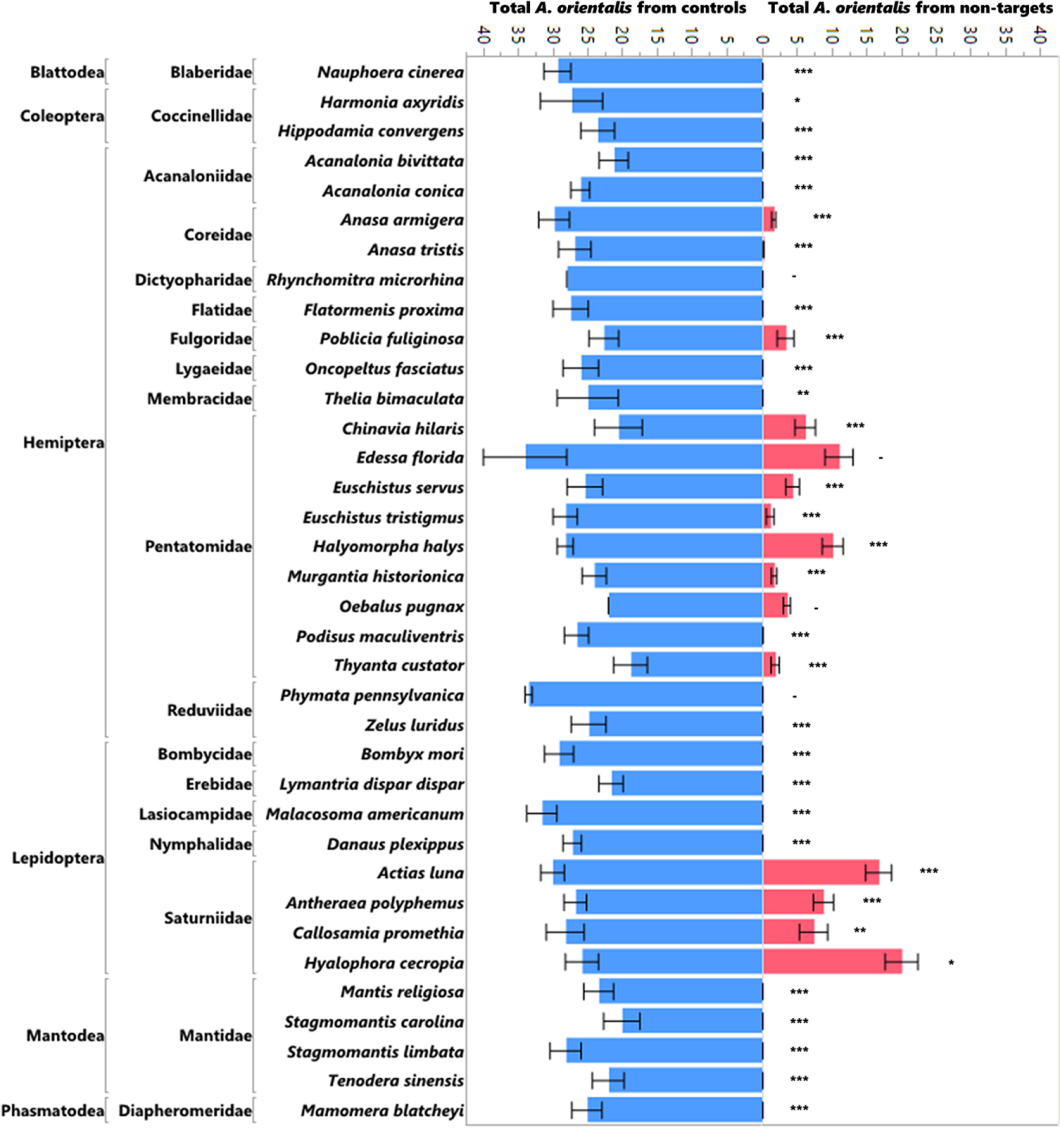
Mean number of *A. orientalis* produced in non-targets (pink) as compared to simultaneously run controls (blue) in no-choice tests organized by insect order and family. The error bars represent standard errors. Wilcoxon paired-sample test; *P < 0.05; **P < 0.01; ***P < 0.001; and a dash when the sample sizes were too small to run the test.

**Figure 2:**
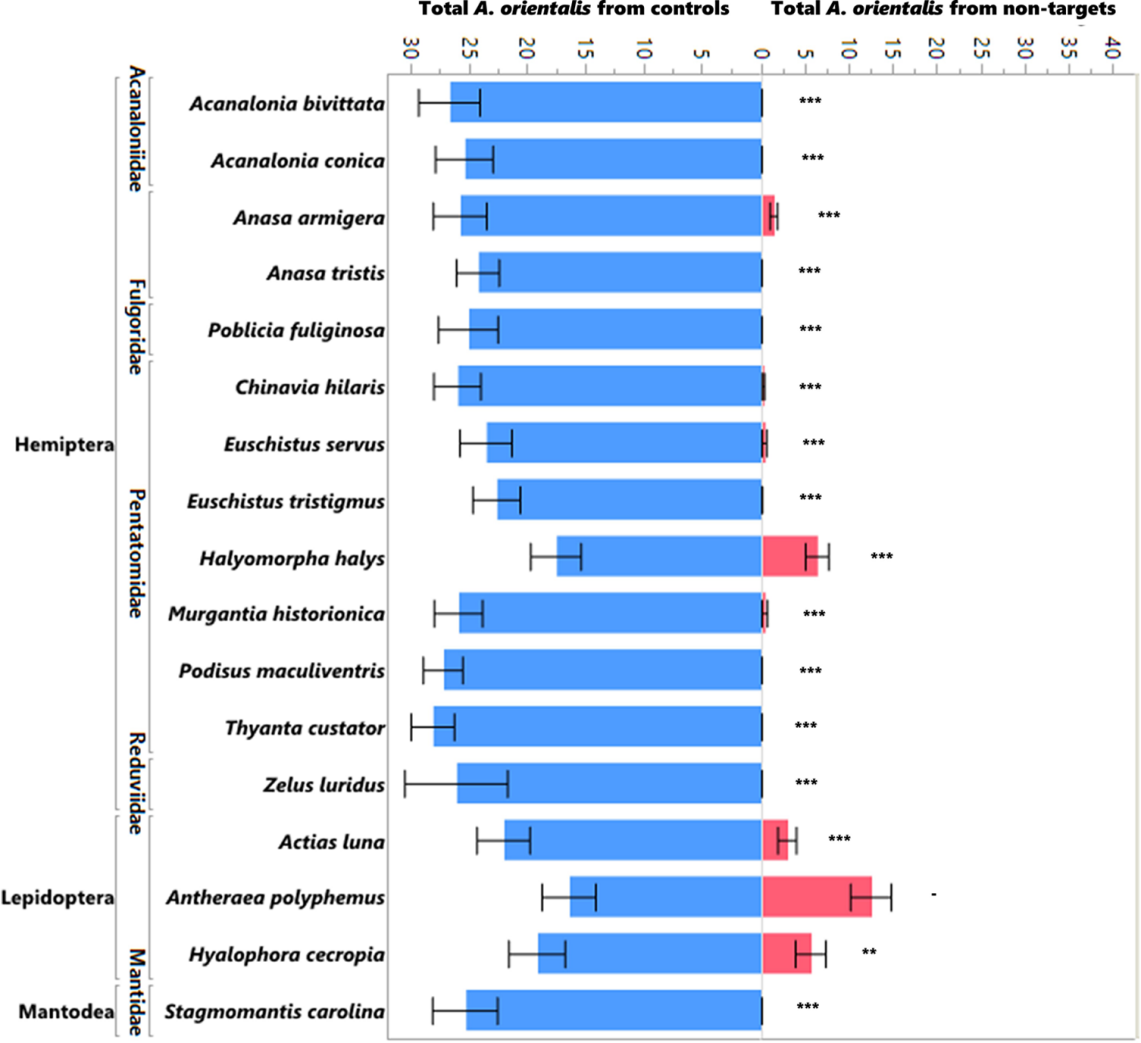
Mean number of *A. orientalis* produced in non-targets (pink) as compared to simultaneously run controls (blue) in choice tests organized by insect order and family. The figure includes all the species that showed attack in the no-choice tests as well as four additional species (*A. bivittata, A. conica, Z. luridus*, and *S. carolina*) that were put into testing. The error bars represent standard errors. Wilcoxon paired-sample test; **P < 0.01; ***P < 0.001; and a dash when the sample sizes were too small to run the test.

The production of progeny on non-target egg masses was significantly lower than the production of progeny on the controls (*L. delicatula* egg masses run simultaneously) in the no-choice tests (**Figure 1**; χ^2^ = 1172.97, df = 1, p<0.0001) as well as in the choice tests (**Figure 2**; χ^2^ = 481.07, df = 1, p<0.0001). Ten of the 16 species that were attacked in the no-choice tests were also attacked in the choice tests (**Table 3**), although two of these (*Euschistus tristigmus* (Say) and *Thyanta custator* (Fabricius)) showed negligible attack rates. The non-target hosts that experienced the highest attack and the closest attack rates to that of the *L. delicatula* controls were the giant silk moths (Saturniidae) eggs, followed by stink bugs (Pentatomidae). For *Edessa florida* Barber, *Oebalus pugnax* (Fabricius), and *Callosamia promethea* (Drury), three species that were attacked in the no-choice tests, we were not able to acquire additional eggs for the subsequent choice testing. However, we conclude from the other tests of stink bug and silk moth eggs that these likely would have been utilized. For four species (*Acanalonia bivittata* (Say)*, Acanalonia conica* (Say)*, Zelus luridus* Stål, and *Stagmomantis carolina* (Johannson)) that were not attacked in the no-choice testing, we had extra available eggs and so we conducted a small number of choice tests with them as well, and no progeny were produced in the choice tests either. The number of progeny produced in the choice testing was overall lower than in the no-choice testing. This was true for the non-target eggs tested as well as the corresponding *L. delicatula* controls, and when more attack on non-targets was evident there was also less attack on the *L. delicatula* controls (as evident in results from testing the saturniids and *H. halys*).

For 29 non-target species, a subset of egg masses was dissected (**Table 2**). We found no unemerged *A. orientalis* in 18 of the species and very little in the rest. In 11 species (*A. luna, Anasa armigera* (Say)*, Anasa tristis* (DeGeer)*, Antheraea polyphemus* (Cramer)*, Chinavia hilaris* (Say)*, Euschistus servus* (Say)*, H. halys, Hyalophora cecropia* (L.)*, Murgantia histrionica* (Hahn)*, P. fuliginosa, T. custator*), all of which also successfully reared some *A. orientalis*, we found low numbers of unemerged *A. orientalis* present in the host eggs, either in a diapause state or as desiccated adults. Due to the scope of these dissections, we did not quantify dead early immature or encapsulated wasp larvae. Proportions of dissected eggs with unemerged *A. orientalis* were low, ranging from 0.01 to 0.21 (i.e., 1 to 21%) of egg masses with *A. armigera* and *T. custator* showing the lowest rates and *A. tristis* the highest rates. For all non-target species egg masses with some unemerged wasps, on average 1.44 ± 0.26 wasps per egg masses did not emerge. Parasitized *L. delicatula* egg masses reared under the same conditions had a similar rate of unsuccessful emergence, a proportion of 0.08 (or 8%) or a mean of 3.05 ± 0.60 wasps per egg mass that did not successfully emerge.

### Effect of egg size

**Figure 3:**
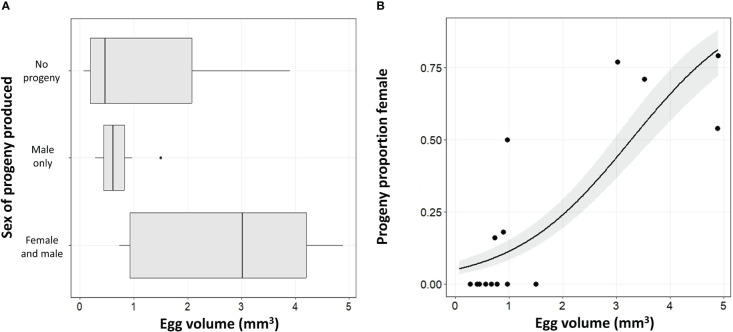
Mean egg volume (mm^2^) and the resulting wasp emergence patterns of the progeny showing **(A)** the sex of the progeny that emerged and **(B)** the proportion female.

The size of the non-target egg presented to the *A. orientalis* wasps was important not only as a factor in whether the egg was successfully used but also in the resulting sex bias. Egg size had a significant effect on the sex of progeny produced (df = 1; χ^2^ = 9.26, p = 0.0023; **Figure 3A**). There was a significant relationship between egg volume and the sex ratio of the progeny produced with larger eggs having a female skewed sex ratio (df = 1; χ^2^ = 8.25, p = 0.0041; **Figure 3B**). The giant silk moth eggs overall showed the highest proportion of female progeny and were the only non-target species that resulted in female progeny in both the no-choice tests and the choice tests (**Table 3**). The giant silk moths also had some of the largest eggs that were put into testing. The species with the next highest ratio of female to male progeny was *P. fuliginosa*, followed by species of stink bugs.

### Fitness of *A. orientalis* reared from non-target eggs

**Figure 4:**
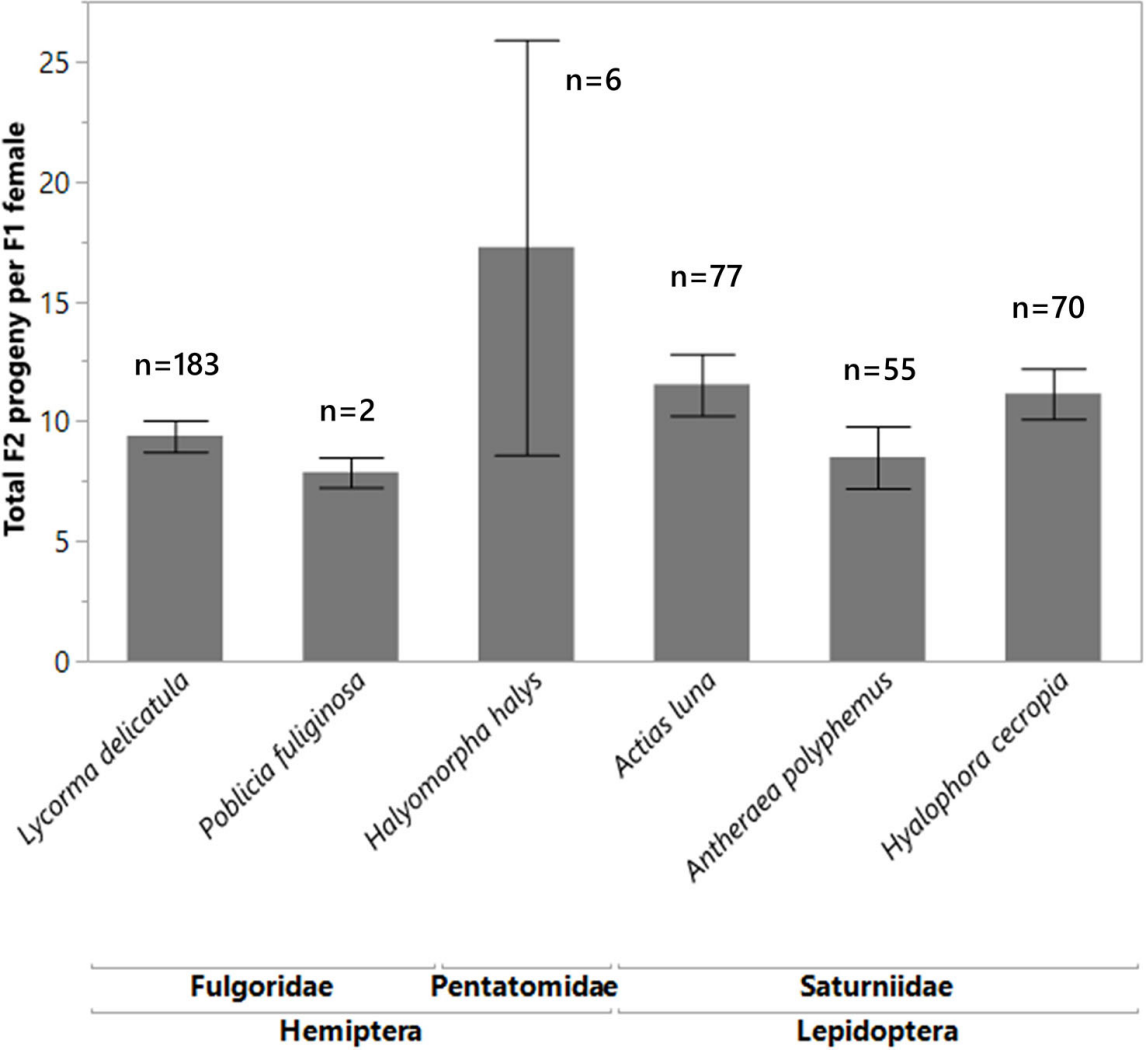
Mean number of F2 progeny wasps produced from spotted lanternfly eggs masses for each F1 female, which was reared from non-target species eggs. There was no significant difference (df = 5, 387, F = 1.6728, p = 0.1401) between the number of progeny produced by females reared out from the non-targets as compared to those reared from *L. delicatula*. The sample sizes are written above each species.

For the non-target species that were attacked, and which resulted in female wasp progeny (F1 generation), the F1 females were able to produce their own progeny (F2 generation) at the same rate as the F1 females that were reared from the control *L. delicatula* eggs. There was no significant difference (df = 5, 387, F = 1.6728, p = 0.1401) between the number of F2 wasp progeny produced by the F1 females reared out from the non-targets as from the F1 females reared out of *L. delicatula* (**Figure 4**). By comparing the mean tibia measurements of female *A. orientalis* wasps (F1 generation) reared on a large non-target host (*A. luna*) and a small non-target host (*H. halys*) compared to controls (*L. delicatula*), we found that the females reared from *H. halys* eggs were significantly smaller (0.59 mm ± 0.02 SE) than those reared from *A. luna* (0.88 mm ± 0.01 SE) and *L. delicatula* (0.94 mm ± 0.01 SE; df = 2, 22, F-ratio = 131.068, p < 0.0001). When these differently sized females, reared from different hosts, were all provided *L. delicatula* egg masses, their female progeny did not show any significant differences in tibia size (df = 2,27, F-ratio = 0.0655, p = 0.9368). Smaller progeny from non-target hosts were able to attack *L. delicatula* at the same rate as larger females and their progeny were of normal size.

## Discussion

Evaluation of the physiological host range is one of the first steps in assessing the risk-benefit potential of a biological control agent to determine if it is sufficiently host-specific (16, 17). The results from this study demonstrate that *A. orientalis* Haplotype C is physiologically able to develop in non-target egg masses of coreids, other fulgorids, pentatomids, and saturniids. However, the number of progeny produced from the non-targets is consistently lower than from the *L. delicatula* controls and the attack rates were particularly low when the wasps had a choice of using the non-target egg masses or the *L. delicatula* egg masses. The fact that we did not see attack on *P. fuliginosa* in the choice tests, even though the no-choice testing tells us that it is physiologically possible is interesting because, of the species tested, *P. fuliginosa* is the one most closely related (and biologically similar) to *L. delicatula*. Additional host range testing of western fulgorid planthoppers, as well as other potential non-targets that are resident to the western United States, was conducted by collaborators at the University of California Riverside (21). Together these studies show that *A. orientalis* Haplotype C prefers to parasitize *L. delicatula* egg masses but is willing to attack and is physiologically able to develop in various non-target species suggesting that it is facultatively oligophagous or polyphagous.

We found that *A. orientalis* Haplotype C is more likely to use large eggs, and more often used these large eggs to produce female progeny, and the progeny from large eggs are larger than those reared from smaller eggs. The three largest eggs included in the study were those of saturniid (giant silk moth) eggs—*A. luna*, *A. polyphemus*, and *H. cecropia*. Along with the two fulgorids— *L. delicatula* and *P. fuliginosa*—these showed the highest levels of attack and the highest proportion of female progeny. This is commonly found with other species of egg parasitoids (30, 31). We did not find any notable differences in the number of developing wasps that did not successfully emerge from the egg masses and the rate of this occurrence was very similar in the non-targets as it was for the controls. While *H. halys* was previously found to not be parasitized by *A. orientalis* (15), our tests and those of our colleagues (21) found that it could be parasitized. Also, Seo et al. (15) found that the saturniid *Antheraea pernyi* was only parasitized if the eggs were immature and dissected out of a gravid female. However, we found that older saturniid eggs were readily parasitized by *A. orientalis.* It may be that this difference in host range is due to differences in the haplotype or strain of *A. orientalis* tested in these studies. Future studies are planned to test the host range of other detected *A. orientalis* haplotypes.

Various factors may have affected our results. One factor could be the number of eggs presented to the wasps for each test. Wasps were given enough eggs to satisfy their oviposition needs. On average there were 42.9 ± 3.0 (mean ± SE, n = 84) *L. delicatula* eggs available in each egg mass tested, from which were produced on average 23.0 ± 0.43 wasp progeny during the one-week exposure period. The non-target replicates had an average of 27.4 ± 0.4 (mean ± SE, n = 725) eggs per replicate. This is a lower number of eggs per replicate than the control *L. delicatula* because they either did not lay their eggs in large masses or were challenging to rear in numbers. Considering that the mean number of adult wasps produced from the *L. delicatula* replicates was 23 wasps/egg mass, the wasps were provisioned with enough eggs such they were unlikely to use all available host eggs by the end of the oviposition period. Contamination by resident *Anastatus* species at the site of *L. delicatula* egg mass collection is another potential factor. However, parasitism of field-collected eggs by *Anastatus* species native or resident to the U.S. has been shown to be extremely rare (unpublished data), and no or at least inconsequential numbers would have been present in egg masses used for this study. The rearing temperatures, Beijing-fall conditions, were selected to optimize *A. orientalis* fitness (20) and are not necessarily the optimal rearing condition for the non-target species. However, for the most conservative tests possible here, we felt it was important to prioritize conditions for the parasitoid rather than for the various hosts. And lastly, when put into testing there was some variability of the age of eggs. Most non-target eggs were tested when they were less than a week old (median age = 6 days old, n=991) however there was variation around this median. The mean age was higher (12.5 ± 0.52 days). When exposed to wasps, the eggs of *A. bivittata*, *A. conica*, *B. mori*, *Flatormenis proxima* (Walker), *P. fuliginosa*, and *Tenodera sinensis* Saussure all had a median age that was greater than a week (13, 20, 49, 21, 30, and 18.5 days, respectively). The eggs of all other species were exposed when less than a week old. The fact that the eggs of these species were overall older may have artificially decreased their attractiveness or viability for *A. orientalis* as has been seen with other egg parasitoids (32–34). Additionally, the control eggs (of *L. delicatula*) were collected over the winter, stored in chill as described above, and used throughout the year whenever any of the non-targets were available for testing. However, based on prior research, *L. delicatula* eggs even up to a year old were viable hosts for *A. orientalis* with no discernable effect on parasitism rate (20).

While no-choice and choice host range testing are essential steps in assessing the risk-benefit of a candidate classical (or importation) biological control agent, it is important to keep in mind that these tests determine the ability of a parasitoid to physiologically use the hosts. By design, these tests are conducted in a controlled laboratory setting, which purposely limit the complexities of environmental conditions. In addition, any research conducted with *A. orientalis* in the U.S. as a candidate agent must be done in quarantine containment. Thus, these tests can overestimate ecologically relative host usage due to the limited ecological and environmental aspects of the tests (35, 36). In a field setting many additional factors influence a natural enemy’s ability to locate and utilize a potential host. A parasitoid must first be able to locate the host within a complex habitat, and once found, it may either accept it or decide to continue searching. For example, *Trissolcus japonicus* (Ashmead), a parasitoid of *H. halys*, displayed an oligophagous physiological host range in laboratory choice and no-choice assays (37–39) but a more restricted behavior in laboratory behavioral assays and field tests (40, 41).

Studies that focus on a natural enemy’s ability to find hosts include, but are not limited to, testing for attraction response to kairomones left by the non-targets and target pest species or large arena studies where the natural enemy is provided a larger and more complex space to search for the target and non-target hosts. Prior studies that tested the foraging behaviors of *A. orientalis* when in the presence of residues left by *L. delicatula* and the oothecal covering of *L. delicatula* eggs found that wasps detected chemical traces left by the *L. delicatula*, eliciting a strong arrestment response (42). Subsequent work evaluating ecological host range found that *A. orientalis* spent significantly more time interacting with chemical traces left behind by *L. delicatula* than the controls or than with the chemical traces left by *P. fuliginosa* (unpublished). This provides additional evidence that the preferred host of *A. orientalis* is *L. delicatula*. Tests evaluating the host range of *A. orientalis* in the field in China are planned using sentinel egg masses.

The population of *A. orientalis* tested in this study was Haplotype C. However, several other haplotypes of *A. orientalis* have been identified and these have now been separated into isofemale lines (19). Laboratory studies evaluating the rearing of the haplotypes shows that they respond differently to rearing conditions (19). This suggests that these haplotypes are genetically distinct but also that their biologies are distinct. Previous host range testing of *A. orientalis* conducted before release of it as a biocontrol agent in South Korea showed that it was highly host specific to *L. delicatula* and that *H. halys* eggs did not support development of *A. orientalis* (15). This is different than the testing results we obtained with Haplotype C, suggesting that the haplotype tested for release in South Korea may have been a different haplotype, potentially Haplotype D (19). Unlike our colony of Haplotype C wasps, the line tested for release in South Korea was successfully reared using constant 25°C temperature and long day light conditions, adding further evidence that what was released in Korea and the strain we are studying have different biological characteristics. Additional host range testing will be conducted with the additional genetic lines we have in colony. However, this study, along with the work of Gómez Marco et al. (21), shows that *A. orientalis* Haplotype C is willing and able to develop in various non-target species but prefers to parasitize *L. delicatula*.

## Data availability statement

The raw data supporting the conclusions of this article will be made available by the authors, without undue reservation.

## Author contributions

HB, SS, DP, KH, SD, JK, and JG designed the study. X-YW and L-MC provided the parasitoids for the study. HB, SS, DP, YW, and SD ran the studies. HB, SS, DP, KH, LT, TH, CB, AR and JK provided non-target insects to test. HB, SS, DP, and YW summarized the results. HB, SS, DP, TH, AR, JK, YW, and JG wrote the manuscript. All authors contributed to the article and approved the submitted version. 
